# Childhood vaccination uptake and associated factors among children 12–23 months in rural settings of the Gambia: a community-based cross-sectional study

**DOI:** 10.1186/s12889-021-11810-9

**Published:** 2021-09-25

**Authors:** Ebrima Touray, Amadou Barrow, Bakary Kinteh, Mansour Badjie, Musa Nget, Jainaba Touray, Sambou L. S. Kinteh, Solomon P. S. Jatta, Lamin Ceesay

**Affiliations:** 1 School of Public Health, Gambia College, Brikama, The Gambia; 2 Regional Health Directorate, Upper River Region, Ministry of Health, Basse Santa Su, The Gambia; 3 Department of Public & Environmental Health, School of Medicine & Allied Health Sciences, University of The Gambia, Kanifing, The Gambia

**Keywords:** Childhood immunization, Vaccination uptake, Caregivers, Health education, The Gambia

## Abstract

**Background:**

Globally, immunization prevents 2–3 million deaths annually from vaccine-preventable diseases such as diphtheria, tetanus, pertussis, influenza, and measles. In developing countries, several immunization programs have made progress, but the coverage remains a standstill in some areas. In order to inform policies and practices, the present study aimed at assessing vaccination uptake and contextual-associated factors among children aged 12–23 months in rural Gambia.

**Methods:**

A community-based triangulated cross-sectional design was conducted in January 2020, with 200 caregivers with children aged 12–23 months in selected households in rural communities across Upper River Region of the Gambia using multistage sampling technique were recruited. A structured interview questionnaire was developed and Infant Welfare Cards were assessed to elicit information regarding contextual household characteristics towards childhood immunization uptake. Percentages, chi-square/fisher exact test for variables with *p*-value ≤0.15 were considered for inclusion into logistic regression model. The significance level was set at *p* < 0.05. The adjusted Odds Ratio (aOR) with 95% Confidence Interval (CI) were reported to declare significance.

**Results:**

The proportion of children who received all the required vaccines was 66%. At the level of antigen-specific coverage, about 88.5% received BCG, 71% received OPV 3, 82.5% received Penta 3, while 72 and 71% received Measles-Rubella and yellow fever, respectively. Caregivers who had primary education level 88.8% (aOR = 0.112; 95% CI = 0.029–0.434), secondary & above 87.2% (aOR = 0.128; 95% CI = 0.029, 0. 561) and arabic/madrassa 95.7% (aOR = 0.043; 95% CI = 0.008–1.227) were less likely to be fully vaccinated when compared to those who have never been to school. Farmers are less likely by 88.9% (aOR = 0.111; 95% CI 0.020, 0.635) while children from family size of more than 20 members had reduced odds (aOR = 0.420; 95% CI = 0.197, 0.894) for their children to complete their vaccination schedule as compared to those with at most 20 household members.

**Conclusion:**

There is moderately a burden of incomplete vaccination in rural Gambia. Vaccination programs should be constantly monitored and evaluated by the Ministry of Health, especially in rural areas. To increase societal awareness and vaccine acceptance, a robust community-based health education efforts are desperately needed as part of initiatives to increase vaccine service utilization for these high-risk classes.

## Background

Since the inception of Expanded Program on Immunization (EPI) by The World Health Organization (WHO) in 1974, immunization has become one of the most successful and cost-effective public health interventions. It was created with the ultimate purpose of vaccinating children throughout the world in the control and prevention of infectious diseases [1, 2]. Over the decades, remarkable achievements have been registered towards developing immunization programmes by saving millions of lives and lifelong disabilities worldwide [3]. Currently, immunization prevents 2–3 million deaths annually from communicable diseases such as diphtheria, tetanus, pertussis, influenza, and measles [4]. In the WHO-AFRO region, the immunization coverage in 2014 was at 77%, with 90% immunization coverage at the national level in up to 18 countries [5]. However, in sub-Saharan Africa, immunization coverage remained at 72% for the past five years and nearly 31 million children under the age of 5 years suffer from vaccine-preventable diseases annually [6].

Several immunization programs have made progress in developing countries, but the coverage remains at a standstill in some areas. A good number of children do not complete their immunization schedule due to certain challenges that span across caregivers, barriers, and other related factors [7]. Studies have reported high awareness level and perceptions of mothers towards childhood immunization [8, 9]. However, a study by Hassan et al. in 2019 [10] reported poor knowledge and perception of mothers towards supplementary immunization activities and showed no significant association between the socio-demographic, socio-economic factors and perception towards supplementary immunization activities. A similar study by Sarfaraz et al. 2017, showed a significant difference in mothers’ knowledge, attitude, and perception towards childhood immunization through counselling as an intervention with a score of 2–4 in pre-intervention to a score of 10–12 in post-intervention [11]. The unavailability of vaccine services and migration of caregivers have led to incomplete immunization of children [12]. Furthermore, mothers educational level, household income level, and trekking distance to the clinic sites as additional factors that hindered coverages [13].

Since the start of EPI services in May 1979, The Gambia has been registering high immunization coverage of over 85% in BCG, the third dose of DPT-Hep B-Hib, and measles vaccine [4, 14]. Despite this high immunization coverage, poor or marginalized communities have continued to register low immunization coverage, which has a near-stagnation on the immunization coverage for the past year to 92 and 93% in 2017 and 2018, respectively [4]. According to The Gambia’s Demographic Health Survey (DHS) 2019–2020, URR has a vaccination prevalence of 78.6% of all age-appropriate vaccinations 12–23 months, 13.6% less compared to the 2013 DHS [15, 16]. Low immunization coverage in developing countries has mostly been associated with socio-economic and demographic factors such as economic status, educational level of caregivers, geographical area, gender, and ethnicity [14]. Thus, this community-based triangulated study aimed at assessing childhood vaccination uptake and contextual-associated factors among children aged 12–23 months in rural Gambia. The study expands the body of knowledge on immunization services in rural areas and guides policymakers in improving the immunization programs as a whole.

## Methods

### Study area

The study was carried out in the Upper River Region (URR) of The Gambia. URR is one of the 5 local administrative regions with its administrative capital at Basse Santa Su. URR has a total population of 237, 220 with a population density of 115.93 persons/sq.km and a total fertility rate of 7.0% [17, 18]. It has an under five population of 11, 861 and under-fiver mortality of 56/1000 live births [15]. It has 15, 975 household in 369 settlements in 7 districts [15]. Most of the people in this area are involved in farming and business. It has one regional level health facility and 159 peripheral health centers [19].

### Study design, population, and selection of participants

A community-based triangulated cross-sectional design was conducted in January 2020. The study was focused on understanding the perception of caregivers on vaccination, challenges faced in vaccinating their children, and the influence of the socio-demographic and proximate factors on vaccination status. Questionnaires were administered to caregivers in each selected households with children aged 12–23 months. A multistage sampling method was used in this study. Phase I: A cluster sampling strategy was used to select two districts out of the seven districts, one from the northern part and the other from the southern part of URR, through simple random sampling. Phase II: At the selected districts, simple random sampling was done to select households in each catchment areas. Phase III: The target population at each of the selected communities in the catchment area was used to determine the number of caregivers to be interviewed. Participants were selected using a simple random sampling method.

### Sample size

A sample size of 200 was estimated using cochran’s formula with a childhood vaccination prevalence of 14. 6% in one of the Local Government Areas for children under 3 years [16], z of 1.96 for 95% confidence level, and sampling error at 5%. However, the researchers adjusted the final sample size to 200 participants.

$$ {n}_o=\frac{z^2(p)(q)}{e^2} $$

### Data collection tools and techniques

Data were collected by trained students at the School of Public Health, Gambia College, using structured questionnaires. The information regarding socio-demographic factors, perception of vaccination, and challenges faced in routine vaccination activities were collected. Some aspects of the questionnaires were adapted from The Gambia Demographic Health Survey 2013, a thorough review of literature, and consultation with experts [15, 20]. The questionnaires were developed in English first, then translated into Mandinka, Fula, Wolof, and Serahule. The respondents were the primary caretakers of the child 12–23 months. Face to face interview was done with the caregivers to collect data. To determine the vaccination status, whether complete or partial, the Infant Welfare Cards were assessed. In a situation where caregivers’ did not have the child’s IWC, they were excluded from the study.

### Study variables

#### Outcome variables

Immunization status of children. This was classified into two categories: “*Fully vaccinated*” a child within 12–23 months who received vaccination against tuberculosis (also known as BCG), three doses of DPT-HepB-Hib (Penta), three doses of polio vaccines, and one dose of vaccination against measles [16]; “*Partially vaccinated*” who missed at least one of any of the doses of the routine vaccines before turning 1 year or within 12–23 months old [15, 16].

#### Independent variable

The socio-demographic characteristics of caregivers include age, educational level, family type, caregiver, monthly income of caregiver, family size, occupation of child’s father, and decision-maker on child’s vaccination. The various proximate factors including aspects of caregiver’s perceptions towards childhood vaccination were also explored. The perception was measured using 9 items which span across participants willingness to vaccinate their child, general perception of vaccines, targeted diseases and side effects and were gauged using 5 likert scales: strongly agree, agree, undecided, disagree and strongly disagree.

#### Ethical consideration

The study protocol was reviewed and ethical clearance was issued by the Gambia College’s Research Committee for the study. Before the commencement of the study, ethical approval was obtained from the URR Regional Health Directorate of the Ministry of Health and the community leaders of sampled communities. The people were sensitized about the nature of the study in their local languages (Mandinka, Fula, Wolof, and Serahule). Participation in the study was entirely voluntary and only those that accept to be part of the study were recruited. A written informed consent form was signed by each participant who accepted to be enrolled in the study.

#### Data analysis

Data entry, cleaning and processing for preliminary data analysis was done using IBM SPSS version 25.0. Descriptive analysis was presented in frequencies, proportions, and graphs to summarize the data. Bivariate analysis using chi-square/fischer exact test as well as binary logistics regression analysis was done to identify the association between independent and dependent variables. The Chi-square/fisher exact test for variables with *p*-value ≤0.15 were considered for inclusion into logistic regression model. The adjusted odds ratios (aORs) and confidence intervals of 95% were calculated. A *p-*value < 0.05 was considered for statistical significance.

## Results

### Socio-demographic characteristics of respondents

In this study, a total of 200 respondents were recruited with an overall mean age of 29.7 ± 7.4 years. The response rate for the study was 100%. The majority of the respondents accounting for 53% were found to be between the ages of 26 and 35 years in the distribution. The entire respondents were female and about 53% had never been to school or attended formal education. Slightly more than half of the respondents (63%) live in an extended family with a family size of less than 20 at 69.5%. In terms of Caregivers’ occupation and that of the child’s father, the majority were involved in farming at 47 and 61%, respectively. Almost half of the respondents (47.5%) earned less than D1,500 per month and about 47% reported being decision-makers regarding the child’s vaccination issues. Factor such as educational level, family size and occupation of child’s father were significantly associated with (*p* < 0.05) as presented in Table 1.

**Table 1:** Socio-demography characteristics of respondents in URR

Variables	N (%)	Vaccination coverage		***P***-value
		Partially vaccinated n (%) 68 (34.0%)	Fully vaccinated n (%) 132 (66.0%)	
**Age of respondents**				0.204
16–25	60 (30)	25 (12.5)	35 (17.5)	
26–35	107 (53.5)	30 (15.0)	77 (38.5)	
36 & above	33 (16.5)	13 (6.5)	20 (10.0)	
**Educational level**				0.001*
Never been to School	106 (53.0)	41 (20.5)	65 (32.5)	
Primary	41 (20.5)	13 (6.5)	28 (14.0)	
Secondary +	19 (9.5)	11 (5.5)	8 (4.0)	
Madarassa	34 (17.0)	3 (1.5)	31 (15.5)	
**Family Type**				0.161
Single parenting	7 (3.5)	5 (2.5)	2 (1.0)	
Nuclear	67 (33.5)	24 (12.0)	43 (21.5)	
Extended	126 (63.0)	39 (19.5)	87 (43.5)	
**Occupation of Caregiver**				0.116
Currently not working	18 (9.0)	11 (5.5)	7 (3.5)	
Farming	94 (47.0)	30 (15.0)	64 (32.0)	
Housewife	72 (36.0)	24 (12.0)	48 (24.0)	
Business	16 (8.0)	3 (1.5)	13 (6.5)	
**Monthly earning of Caregiver**				0.448
Less than D1500	95 (47.5)	37 (18.5)	58 (29.0)	
D1500 - D3500	60 (30.0)	15 (7.5)	45 (22.5)	
More than D3500	45 (22.5)	16 (8.0)	29 (14.5)	
**Family Size**				0.035*
1–20	139 (69.5)	54 (27.0)	85 (42.5)	
More than 20	61 (30.5)	14 (7.0)	47 (23.5)	
**Occupation of Child’s Father**				0.037*
Currently not working	18 (9.0)	10 (5.0)	8 (4.0)	
Farming	122 (61.0)	34 (17.0)	88 (44.0)	
Civil Servant	7 (3.5)	3 (1.5)	4 (2.0)	
Business	53 (26.5)	21 (10.0)	32 (16.0)	
**Decision maker on Child’s vaccination**				0.200
Mother/Caregiver	94 (47.0)	36 (18.0)	58 (29.0)	
Father	32 (16.0)	8 (4.0)	24 (12.0)	
Both	74 (37.0)	24 (12)	50 (25.0)	

*Statistical significance p < 0.05

### Overall vaccination coverage and antigen level coverage

**Fig. 1:**
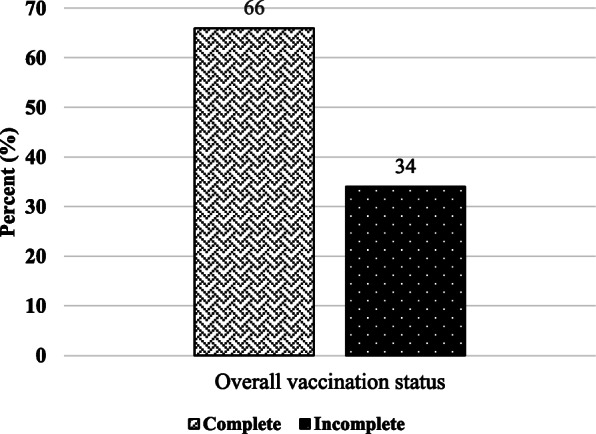
showing overall vaccine coverage

The total proportion of children who received all the required vaccines was 66% while 34% were found to be incompletely vaccinated as shown in Fig. 1.

**Fig. 2:**
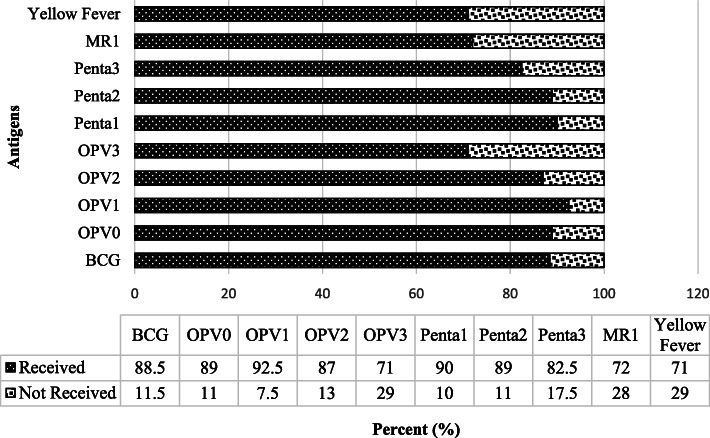
showing antigen level coverage

At the level of antigen-specific coverage, about 88.5% received BCG, 71% received OPV 3, 82.5% received Penta 3, while 72 and 71% received Measles-Rubella and Yellow fever, respectively as shown in Fig. 2.

### Perception of caregivers on vaccination

Of the 200 Caregivers interviewed, 94.5% wanted their children to be vaccinated, and 77.0% believed that their children should be vaccinated, as shown in Table 2. When asked if vaccination can prevent childhood illness, 95% reported that vaccination could prevent childhood illness. More than two-thirds of the respondents (74.0%) believe that vaccines are not harmful. When asked to name the disease conditions that vaccination prevents, the majority mentioned tuberculosis at 76.5%, pneumonia at 74.0%, poliomyelitis at 66.5% and rotavirus at 62.5%. Regarding the importance of routine vaccination and campaigns, 95.5% revealed that routine vaccinations and campaigns are important, while about 88.5% indicated that their children got vaccinated during those campaigns.

**Table 2:** Perception of Caregivers on vaccination and its uses

Perception items	Frequency (n)	Percent (%)
**Wants my child to be vaccinated**		
Yes	189	94.5
No	11	5.5
**Vaccination can prevent childhood illness**		
Yes	191	95.5
No	9	4.5
**Should your child be vaccinated**		
Yes	154	77.0
No	46	23.0
**Vaccines are harmful**		
Yes	52	26.0
No	148	74.0
Diseases vaccine prevents*		
Tuberculosis	153	76.5
Diptheria Pertusis Tetanus (DPT)	69	34.5
Measles-Rubella	111	55.5
Hepatitis B	90	45.0
Pneumonia	148	74.0
Meningitis	115	57.5
Poliomyelitis	133	66.5
Rota Virus	125	62.5
Yellow fever	103	51.5
**Children vaccinated during campaigns**		
Yes	177	88.5
No	23	11.5
**Routine vaccinations and campaigns are important**		
Yes	191	95.5
No	9	4.5

*Multiple responses

The study revealed that most caregivers strongly agree that vaccinating their child is necessary with mean of 4.5; all vaccine-preventable diseases are severe (mean 3.9), and all EPI targeted diseases have drugs for treatment (mean 3.8). Poliomyelitis could result in paralysis or even death (mean 3.8) as shown in Table 3.

**Table 3:** Perception of Caregivers on child vaccination

Perception item	N	Mean	Std. Dev	Strongly Agree n(%)	Agree n(%)	Undecided n(%)	Disagree n(%)	Strongly Disagree n(%)
It is necessary to vaccinate a child	200	4.5	0.68	41(20.5)	76(38.0)	32(16.0)	32(16.0)	19(9.5)
Your child will not get Measles if it strikes	200	3.6	1.25	49(24.5)	58(29.0)	34(17.0)	49(24.5)	10(5.0)
If a family member has Pneumonia your child will be infected	200	3.5	1.13	34(17.0)	21(10.5)	31(15.5)	58(29.0)	56(28.0)
Poliomyelitis can cause paralysis and result to death	200	3.8	0.97	55(27.5)	83(41.5)	43(21.5)	11(5.5)	8(4.0)
All the vaccince preventable diseases are severe	200	3.9	0.93	57(28.5)	91(45.5)	34(17.0)	16(8.0)	2(1.0)
All EPI targeted diseases have drug for treatment	200	3.8	1.02	51(25.5)	69(34.5)	62(31.0)	15(7.5)	3(1.5)
Incomplete vaccination is the same as complete vaccination	200	2.6	1.43	33(16.5)	86(43.0)	39(19.5)	29(14.5)	13(6.5)
A child with common cold should be vaccinated	200	3.4	1.24	44(22.0)	87(43.5)	24(12.0)	24(12.0)	21(10.5)
A child with fever should not be vaccinated	200	3.4	1.25	119(59.5)	76(38.0)	0(0.0)	3(1.5)	2(1.0)

### Association between vaccination status and some proximate factors

Table 4 shows a significant association between child’s vaccination status with women’s total number of children and ever cancelled RCH clinic before at *p* = 0.009 and *p* = 0.036, respectively.

**Table 4:** Participants vaccination status and some proximate factors in URR

Variables	Overall vaccination coverage		***P***-value
	Partially vaccinated n (%)	Fully vaccinated n (%)	
**Rating vaccination service providers at a health facility**			0.485
Very Unfriendly	26 (32.5)	54 (67.5)	
Unfriendly	38 (38.4)	61 (61.6)	
Undecided	3 (23.1)	10 (76.9)	
Friendly	1 (16.7)	5 (83.3)	
Very friendly	0 (0)	2 (100.0)	
**Women total number of children**			0.009*
< 5	40 (45.5)	48 (54.5)	
5 to 10	28 (25.2)	83 (74.8	
> 10	0 (0.0)	1 (100.0)	
**Transportation difficulties to the vaccination site**			0.644
Yes	24 (32.0)	51 (68.0)	
No	44 (35.2)	81 (64.8)	
**The convenience of vaccination timing**			0.143
Yes	54 (37.0)	92 (63.0)	
No	14 (25.9)	40 (74.1)	
**Busy schedules allow attending vaccination sessions**			0.476
Yes	34 (36.6)	59 (63.4)	
No	34 (31.8)	73 (68.2)	
**Child’s sickness prevents attending RCH clinic**			0.238
Yes	12 (26.7)	33 (73.3)	
No	56 (36.1)	99 (63.9)	
**Ever canceled RCH clinic before**			0.036*
Yes	20 (47.6)	22 (52.4)	
No	48 (30.4)	110 (69.6)	
**Ever prevents your child from taking vaccination based on rumor**			0.229
Yes	10 (45.5)	12 (54.5)	
No	58 (32.6)	120 (67.4)	
**Fear of vaccine side effects prevents you from taking your child to the RCH clinic**			0.409
Yes	5 (45.5)	6 (54.5)	
No	63 (33.3)	126 (66.7)	
**Attending pre-clinic sessions on vaccination**			0.437
Every time	19 (30.2)	44 (69.8)	
Sometimes	49 (35.8)	88 (64.2)	
**Waiting hours before your child gets vaccinated**			0.873
< 1 h	26 (36.1)	46 (63.9)	
1 - 2 h	27 (32.1)	57 (67.9)	
> 2 h	15 (34.1)	29 (65.9)	

*Statistical significance p < 0.05

### Binary logistics regression model for predicting the association between vaccination status and socio-demographic characteristics

As shown in Table 5, the variables in the model accounted for between 17.3–23.9% of the variation observed in the outcome variable (fully vaccinated status). The model predicted that respondents who had primary education had reduced odds for their children complete vaccination schedule compared with those those who have never been to school (aOR = 0.112; 95% CI = 0.029–0.434) after adjusting for other confounding factors such as occupation of caregivers, family size and occupation of child’s father. There are reduction in the odds of caregivers with secondary & above (aOR = 0.128; 95% CI = 0.029, 0. 561), and arabic/madrassa education (aOR = 0.043; 95% CI = 0.008–1.227), for their children to to complete their vaccination schedules when compared to those who have never been to school after controlling for other confounders. Farmers among the caregivers had reduced odds (aOR = 0.111; 95% CI 0.020, 0.635) for their children to complete the vaccination schedule as opposed to those that are not working at the time of the study when controlled for other covariates. Children from household with family size of more than 20 members had reduced odds (aOR = 0.420; 95% CI = 0.197, 0.894) for their children to complete their vaccination schedule when in comparison with children from households with at most 20 members after adjusting for other confounding factors such as education, occupation of caregivers and that of child’s father.

**Table 5:** Association between participants overall vaccination status and selected socio-demographic variables in URR

Variables	Adjusted Odd Ratio 95% C.I.
**Educational level**	
None (Ref)	1
Primary	0.112 (0.029–0.434)*
Secondary	0.128 (0.029–0.561)*
Madarasa	0.043 (0.008–0.227)*
**Occupation of caregiver**	
Currently Not Working (Ref)	1
Farming	0.111 (0.020–0.635)*
Housewife	0.242 (0.053–1.100)
Business	0.230 (0.049–1.079)
**Family size**	
1–20 (Ref)	1
More than 20	0.420 (0.197–0.894)*
**Occupation of Child’s Father**	
Currently not working (Ref)	1
Farming	0.695 (0.208–2.326)
Civil Servant	2.084 (0.970–4.476)
Business	0.351 (0.045–2.763)

*Statistical significance p < 0.05

*Ref* Reference category, *C.I* Confidence Interval.

## Discussion

Considering the WHO vaccination schedule, a full vaccination uptake among children 0–24 months in rural Gambia was low at 66%. There are variations of coverage across regions, especially when compared to urban areas in the Gambia. These could be attributed to cultural receptivity towards childhood immunisation and potential inequalities in access to immunisation programs [21]. In rural areas of the Gambia, the patriarchal and low literacy level played an important role in influencing vaccination coverage. The available literature revealed that people concern about the child’s safety regarding vaccination had affected coverage, especially in rural settings [22].

In term specific antigens coverage, there are high uptake for OPV 1, pentavalent 1 and 2, while a decline was observed for others such as OPV3, yellow fever and measles-rubella. The current observed trend could be attributed to vaccine hesitancy as a proxy to factors influencing incomplete vaccination. This may be linked to vaccine hesitancy in developed countries due to cultural myths, adverse vaccine effects, and associated consequences [23, 24]. Furthermore, it is also documented that a responsive approach towards the management of adverse events following immunisation in both routine and supplementary immunisation activities could be attributed to parents not allowing their children to be vaccinated. Some vaccines such as measles and pentavalent, had some reactions in a form of abscess at the injection site, fever and irritability [25, 26]. Vaccinating children often protects them from an unknown risks. Since these advantages are not readily evident, there is almost no encouragement for child caregivers to prioritize vaccination programs in the face of conflicting demands for their resources [27].

At least seven in ten of all children received BCG and OPV 3 vaccinations at the time of the study and about seven in ten children received yellow fever and measles-rubella before their first birthday. The study identified factors such as women with any education were less likely to get their children fully vaccinated; families with more than 20 members and women that are farmers were negatively associated with the updake of vaccination in rural Gambia. Contextual factors such as the increased number of children in households, nonliterate caregivers, high illiteracy among the child’s father, or parents who were farmers, incomplete vaccination were found to be more common [26, 28]. Vaccinations were more likely to be incomplete in children with multiple siblings. Larger families tend to put conflicting demands on mothers, restricting the time and resources available to care for each child. Other studies have identified this link, which has been attributed to the higher cost and demands on services caused by having more children in a family, which could have a negative impact on healthcare utilization [29, 30]. According to some reports, the uptake of child vaccination services is linked to maternal education, antenatal care participation, and parity [26, 31, 32]. Furthermore, paternal literacy, which has been used in the Gambia as a surrogate indicator of socioeconomic status, tends to be inversely linked to inadequate immunisation. Similar studies have reported this association [31, 33–35].

If nothing is done about the delay in obtaining vaccines, a pool of children with incomplete immunisation may increase over time [32]. The existence of such a large group of vulnerable children makes outbreaks of vaccine-preventable diseases more likely [36]. Vaccinating children at an early age has institutional, programmatic, and financial implications [32, 37]. The current Gambian immunisation schedule provides additional vaccines such as pneumococcal, rotavirus and human papillomavirus vaccines to cover more vaccine preventable diseases across the country. The advent of emerging social myths regarding the CoVid-19 vaccines have some tendency of making vaccination programmes a challenging tasks for developing countries, including the Gambia. Thus, assessing the completion of vaccinations on time is even more critical for the EPI programme’s success.

Nonetheless, optimistic attitudes about vaccines did not seem to impact the vaccination of children in our sample substantially. In this study, most women (caregivers) desired and made sure their children were fully vaccinated, including those carried out during campaigns. According to this survey, parents strongly believe that vaccinations are essential for their children’s wellbeing, with the majority of respondents holding views that seem to favor immunisation. Children born to younger mothers, those with higher birth orders, and those from larger families have historically been shown to receive less health care services in general and preventive services in particular [35, 38–41]. Slightly less than half of them have the decision-making powers regarding their child’s utilisation of vaccines. As a result, these create rooms for the cancellation of some RCH clinics where children are administered vaccines. These may be attributed to the biased-power dynamics against women, especially those who are majorly never been to school and engaged as farmers. It could mean that ANC services are well attended and could be a proxy to predict institutional deliveries reported from other studies [29, 42–44].

### Study limitations

This research was carried out in the communities of URR, Gambia. As a result, our findings may have implications for vaccination programs in Gambia and, by extension, West Africa. Respondents who did not have Infant Welfare Cards were excluded from the sample, which may have resulted in distorted sampling. People without IWCs were likely to have only partially immunised their children, never received, or had more delayed vaccines than those with cards. Additionally, vaccine experiences may have affected certain caregivers’ beliefs, views, and attitudes. As a result, vaccination coverage in rural Gambia may be lower than what is recorded in this research. Exploring other potential and inventive means of reliable data sources for immunisation programmers will be one of the issues for additional research on childhood immunisation coverage. Furthermore, we recommend a follow-up study to understand the biased power dynamics against women in terms of decision making for children’s vaccination uptake in rural and urdan areas.

## Conclusion

Our analysis shows that a substantial number of children completed their vaccination as expected. There is moderately a burden of incomplete vaccination in rural Gambia. This burden is heavily influenced by factors such as the caregiver’s educational level, occupation, family size, and father’s occupation. Vaccination programs should be constantly monitored and evaluated by the Ministry of Health, especially in rural areas. To increase societal awareness and vaccine acceptance, related initiatives such as ANC programs, nutritional surveillance, and postnatal care services must be strengthened and expanded. Strong community-based health education efforts are desperately needed as part of initiatives to increase vaccine service utilization for these high-risk classes.

## Data Availability

The data used to support the findings of this study are available upon reasonable request from the school administration at sph@gambiacollege.edu.gm.

## Abbreviations

aOR: Adjusted odds ratio
BCG: Bacille Calmette-Guerin
DHS: Demographic Health Survey
DPT-HepB-Hib: Diphtheria, Pertussis, Tetanus, Hepatitis B, and Hemophilus influenza type B (Penta)
EPI: Expanded Program on Immunization
IWC: Infant Welfare Card
SPSS: Statistical Package for the Social Sciences
URR: Upper River Region
WHO: World Health Organization
